# Discrimination of New and Aged Seeds Based on On-Line Near-Infrared Spectroscopy Technology Combined with Machine Learning

**DOI:** 10.3390/foods13101570

**Published:** 2024-05-17

**Authors:** Yanqiu Zhu, Shuxiang Fan, Min Zuo, Baohua Zhang, Qingzhen Zhu, Jianlei Kong

**Affiliations:** 1 Key Laboratory for Theory and Technology of Intelligent Agricultural Machinery and Equipment of Jiangsu University, Zhenjiang 212013, China; 1000004953@ujs.edu.cn; 2 College of Technology, Beijing Forestry University, Beijing 100083, China; fanshuxiang@outlook.com; 3 National Engineering Research Center for Agri-Product Quality Traceability, Beijing Technology and Business University, Beijing 100048, China; zuomin@btbu.edu.cn; 4 College of Artificial Intelligence, Nanjing Agricultural University, Nanjing 210095, China; bhzhang@njau.edu.cn

**Keywords:** maize seeds, on-line assessment, NIR spectral, effective wavelength selection

## Abstract

The harvest year of maize seeds has a significant impact on seed vitality and maize yield. Therefore, it is vital to identify new seeds. In this study, an on-line near-infrared (NIR) spectra collection device (899–1715 nm) was designed and employed for distinguishing maize seeds harvested in different years. Compared with least squares support vector machine (LS-SVM), k-nearest neighbor (KNN), and extreme learning machine (ELM), the partial least squares discriminant analysis (PLS-DA) model has the optimal recognition performance for maize seed harvest years. Six different preprocessing methods, including Savitzky–Golay smoothing (SGS), standard normal variate transformation (SNV), multiplicative scatter correction (MSC), Savitzky–Golay 1 derivative (SG-D1), Savitzky–Golay 2 derivative (SG-D2), and normalization (Norm), were used to improve the quality of the spectra. The Monte Carlo cross-validation uninformative variable elimination (MC-UVE), competitive adaptive reweighted sampling (CARS), bootstrapping soft shrinkage (BOSS), successive projections algorithm (SPA), and their combinations were used to obtain effective wavelengths and decrease spectral dimensionality. The MC-UVE-BOSS-PLS-DA model achieved the classification with an accuracy of 88.75% using 93 features based on Norm preprocessed spectral data. This study showed that the self-designed NIR collection system could be used to identify the harvested years of maize seed.

## 1. Introduction

Maize (*Zea mays* L.) is one of the indispensable food crops for human beings and is extensively cultivated worldwide. Maize is a vital raw material for the chemical and medical and health industries, as well as an essential feed source for animal aquaculture and husbandry [1,2]. Moreover, maize has various biological activities [3]. The quality of seed is crucial to agricultural production. High-quality maize seeds not only increase yields and ensure the consistency of plant growth, but are also conducive to using drones to conduct emasculation, pesticide spraying, and other operations [4]. The quality of seed can be determined by its physicochemical properties or germination ability. The nutrition of the maize seeds will be lost with the extension of the storage time, resulting in a low germination rate and weak seedlings [5]. The newly harvested seeds have high vigor, presenting a high germination rate. Therefore, the seedlings of high-quality seeds tend to be strong and healthy.

The traditional methods for inspecting the freshness of maize seeds include manual observation and chemical analysis [6]. The former requires observing the glossiness of the seed surface, while the latter requires soaking the maize seeds in red ink solution and examining the color of the embryo. However, these methods have disadvantages such as being time-consuming and susceptible to subjective influence, making them unsuitable for rapid maize seed assessment. The promotion of single seed sowing technology in China has put forward higher requirements for seed quality. In order to provide high-quality seeds for cultivation, it is necessary to develop a non-destructive, accurate, and rapid method for identifying aged maize seeds before seeding. Therefore, the technique of the on-line assessment of maize seed, which is used for evaluating and sorting maize seed according to quality at high speed, has enjoyed a great deal of attention from international machinery companies and researchers.

With the storage time extension, the internal protein, starch, fat, and cellulose components in seeds will change, resulting in the decline of the gemination rate. These substances are rich in hydrogen-containing groups such as C-H, O-H, and N-H [7], which can be characterized and identified by spectral technologies. Hyperspectral imaging technology has been widely used in seed quality assessment [8,9,10]. Ambrose et al. [11] used hyperspectral imaging technology to distinguish aged and normal corn seeds. The results showed that the classification accuracy for the calibration and prediction set were 97.6% and 95.6%, respectively. Zhou et al. [12] extracted visible and near-infrared spectral information from hyperspectral images of nine varieties of sweet corn seeds, which were used to build seven classifiers. The support vector machine SVM model developed using feature wavelengths selected by competitive adaptive reweighted sampling (CARS) obtained the optimal classification results with accuracies of 94.86% and 94.07% for germ and endosperm sides, respectively. Zhang et al. [13] applied the fused features of near-infrared hyperspectral imaging to establish classification models to identify the varieties of coated maize kernels and achieved a classification accuracy of over 90%. Wang et al. [6] identified maize seeds harvested in different years by using spectra from the endosperm side, embryo side, and both sides. Fan et al. [14] constructed a YOLOv7 model based on the spectral and image information of maize seeds, obtaining a classification accuracy of 99.7% for maize seed germination. The studies stated above indicated that hyperspectral imaging technology is a powerful tool for maize seed quality assessment. However, the time-consuming acquisition of hyperspectral images with high dimensionality and the high cost of spectral cameras pose great challenges for fast and real-time assessment, which has hindered the development of on-line inspection systems. Therefore, hyperspectral imaging technologies have mostly been used in lab conditions for fundamental research.

In comparison to hyperspectral imaging technology, the advantages of traditional NIR spectroscopy technology are convenience, low cost, high inspection efficiency, and multi-index measurement capability [15]. Recent improvements in miniaturization and mathematical tools has led to a wide application of NIR spectroscopy technology in the quality analysis of agricultural products, such as fruit and seeds [16]. In terms of fruit quality evaluation, NIR spectroscopy technology has been used for fruit sorting by detecting fruit sugar content and internal bruising in a commercial packing line with a speed of five or more fruits per second. For seed quality assessment, Wang et al. [17] used a self-designed NIR spectral collection device to acquire near-infrared spectra of normal maize seeds, artificially aged maize seeds, and heat-damaged maize seeds, which were utilized to establish a classification model using a partial least squares discriminant analysis (PLS-DA) algorithm. The classification accuracy of the prediction set was higher than 95%. Liu et al. [18] built a single-fiber spatially resolved device using visible/near-infrared spectroscopy to inspect maize seed vigor and achieved good prediction results. However, the studies stated above still involve the static assessment of maize seeds. Up to now, far too little attention has been paid to the on-line application of NIR spectroscopy for seed inspection. In a recent study, Wang et al. [19] applied self-designed on-line NIR spectroscopy equipment to evaluate insect-infested seeds and achieved a classification accuracy of 0.89 and 0.83 using full wavelengths or feature wavelengths, respectively. In addition, the protein content of maize seeds was predicted precisely, with an RPD of 2.08 and 2.11 for grain protein content and absolute grain protein content prediction, respectively. Similar results were reported for soybean seeds [20]. These previous studies demonstrated the enormous potential of NIR spectroscopy technology for the on-line assessment of maize seed quality.

Therefore, the aim of this study was to use NIR spectroscopy technology for the on-line discrimination of maize seeds harvested in different years. The specific objectives were (1) to acquire the near-infrared spectra of maize seeds using an on-line NIR device; (2) to establish classification models to identify the harvest year of maize seeds; and (3) to optimize the model using wavelength selection methods.

## 2. Materials and Methods

### 2.1. Preparation of Samples

JINGKE 968 is one of the high-quality varieties of maize seeds that has been widely planted in China. The JINGKE 968 maize seeds were harvested in 2018 and 2021 in the Gansu province of China. The seeds were sent to the lab and placed under room conditions with 60% relative humidity and a temperature of 24 °C. A total of 200 seeds, with 100 for each harvest year, were selected randomly to conduct standard germination tests at 25 °C following the guidelines of the International Seed Testing Association (ISTA) before the experiment. The normal seedlings were counted after 7 days, and the germination rate of the maize seeds harvested in 2018 and 2021 were 76% and 91%, respectively. A total of 400 maize seed samples without surface defects were then utilized in this study in January 2022; half of the samples were harvested in 2018, and the rest were harvested in 2021.

Table 1 shows the partition of the sample sets for establishing classification models. The training set consisted of 320 maize seeds that were selected randomly, including 160 samples harvested in 2018 and 160 samples harvested in 2021. The prediction set consisted of 80 samples, and the number of maize seeds harvested in 2018 and harvested in 2021 was 40, respectively. In addition, a label value (harvested in 2018 = −1 and harvested in 2021 = 1) was assigned to each sample.

### 2.2. Spectral Data Acquisition

**Figure 1:**
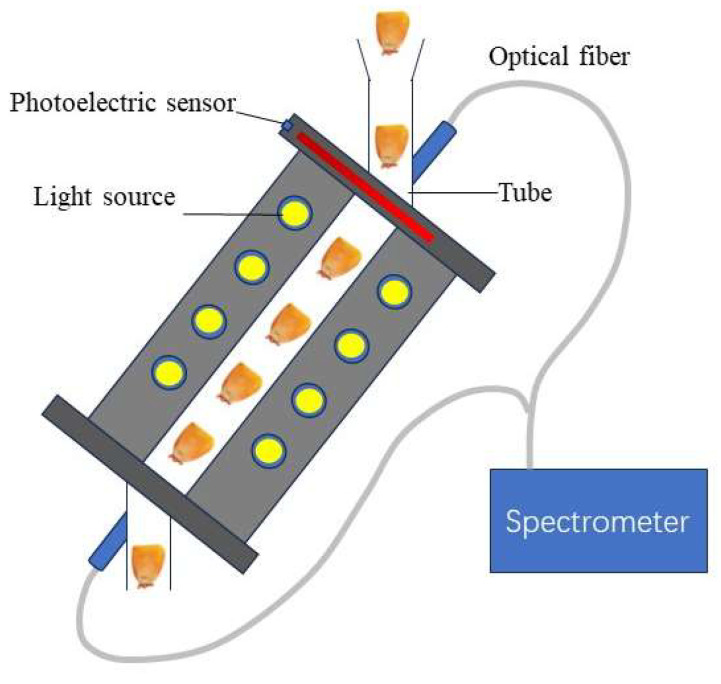
Schematic of the on-line spectra acquisition device.

The NIR spectra were collected using an on-line spectra acquisition device (Ambrose, 2016) (Figure 1). The on-line NIR spectra acquisition device was composed of five parts: an optical fiber, a photoelectric sensor, a light source, a glass tube, and a spectrometer (Ocean optics, Orlando, FL, USA, NIR QUEST, spectra range of 899–1715 nm). The specific process of data collection is described as follows: a single seed triggers the photoelectric sensor as it slides down the tube, which then sends a signal to the spectrometer to collect a spectrum (Sraw) using a specific integration time. The spectrum is collected as the kernel travels down the tube and is displayed and stored in the host computer. Data collection is carried out through a preset integration time. The integration time in the preliminary experiment was set to 100 ms after many attempts.

Typically, the spectral data need to be corrected. In this experiment, data correction was conducted according to the formula below:

(1) $$S_{C}=\frac{S_{raw}}{S_{white}}$$

where *S_c_* was the corrected spectra, and *S_white_* was the white reference acquired by turning on the light source together with no seed in the tube.

### 2.3. Pretreatment of Spectra

The acquired spectral signal not only includes effective chemical information reflecting the samples’ attributes, but also contains noise generated by the measurement environment and equipment. Thus, it is necessary to improve the quality of the raw spectral data before modeling [21]. In this study, six commonly used preprocessing methods were applied for data processing, including Savitzky–Golay smoothing (SGS), standard normal variate transformation (SNV), multiplicative scatter correction (MSC), Savitzky–Golay 1 derivative (SG-D1), Savitzky–Golay 2 derivative (SG-D2), and normalization (Norm). SGS can reduce noise by averaging several spectral points and retaining important features of the spectral curve [22]. The SNV and MSC can eliminate the influence of scattering from the sample surface on the spectral information. SG-D1 derivative with second-order filtering and a smoothing window of 11 points, and SG-D2 with a points second-order filtering smoothing window of 25 were used to identify the overlapping peaks of the original spectra and to reduce interference [23]. For the SGS method, a smaller window size could better preserve spectral features, but may not significantly decrease noise. In contrast, a larger window size is likely to blur significant spectral features during noise elimination. The smooth window was set to 9 after previous experience and many attempts. The Norm method normalizes the data using the Euclidean norm, and can reduce spectral differences caused by slight optical path variations [24]. Therefore, the performance of the above pretreatment methods was compared by evaluating the classification results of the built models.

### 2.4. Effective Wavelength Selection Algorithm

The spectral data, after preprocessing, contains a remarkable amount of uninformative and redundant spectral variables. In addition, there is multicollinearity between the informative variables. The effective wavelength selection method can reduce data dimensions and preserve important information, thereby improving model performance. In this study, four wavelength selection procedures including bootstrapping soft shrinkage (BOSS), CARS, Monte Carlo cross-validation uninformative variable elimination (MC-UVE), and the successive projections algorithm (SPA) were adopted. Moreover, the combination of MC-UVE-BOSS and MC-UVE-SPA algorithms has been proven to be a powerful tool and was consequently also used [25]. 

MC-UVE is a modified variable selection method proposed based on the MC (Monte Carlo) and UVE methods, and is commonly used to eliminate irrelevant information variables [26]. In the MC-UVE, the effectiveness of each variable was evaluated based on its stability defined as the ratio of the mean to the standard deviation of regression coefficients, which was calculated from PLS-DA models based on N subsets randomly sampled using the Monte Carlo approach. The specific details of the MC-UVE algorithm can be referred to in Cai’s paper [27]. The performance of the MC-UVE method is superior to the original UVE method, which has been proven in some previous studies. However, the variables selected by MC-UVE are still redundant to a certain extent; it is therefore necessary to further extract effective variables by combining the approach with other algorithms.

BOSS is a novel variable selection method for reducing the collinearity of spectral data [28], which is derived from the idea of model population analysis and weighted bootstrap sampling. The weight of the variables is obtained by the absolute value of the regression coefficient of numerous PLS sub-models. The weights of variables were updated stepwise using the weighted bootstrap sampling method, resulting in a soft shrinkage of variables. The best variable set is then determined by the sub-model with minimum prediction error.

The CARS algorithm was proposed to eliminate the influence of uninformative variables and improve the performance of the established model [29]. In this algorithm, the absolute values of regression coefficients of the PLS model were used for evaluating the importance of each variable. This algorithm starts by developing a PLS-DA model with full wavelengths. The importance of each variable is evaluated by calculating the absolute values of regression coefficients at each iteration, followed by the elimination of variables using adaptive reweighted sampling and the exponential decreasing function. At each iteration, classification performance is evaluated using a subset of variables at each iteration; the subset with the lowest classification accuracy is determined as containing the best variables. For the CARS effective variables selection, the number of Monte Carlo sampling was set to 100, and 10-fold cross-validation was used. 

SPA is mostly used to eliminate redundant information and reduce the collinearity of spectral data [30]. The SPA algorithm has two phases. First, one wavelength is selected, and its projection on the remaining wavelengths is calculated during each cycle. The wavelength with the maximum projection value is selected as the prospective effective wavelength. Then, the optimal wavelength can be selected based on the smallest RMSECV of the MLR model. Using SPA alone is usually not very effective, so it needs to be combined with other wavelength selection algorithms. 

Combination wavelength selection algorithms were also considered in this study. Taking the example of MC-UVE-SPA, MC-UVE was firstly used to select a set of potential wavelengths, followed by further wavelength extraction using SPA. MC-UVE-BOSS is another combination method that uses MC-UVE and BOSS sequentially for selecting the effective wavelengths.

### 2.5. Model Construction

To gain a reliable and accurate classifier, four classification models, including least squares support vector machine (LS-SVM), partial least squares discriminant analysis (PLS-DA), k-nearest neighbor (KNN), and extreme learning machine (ELM), were built and compared. 

LS-SVM is an evolutionary algorithm based on the standard SVM proposed by Suykens and Vandewalle [31], and is capable of quickly resolving linear and nonlinear multivariable analysis [32]. Compared with standard SVM, it maps input features into a high-dimensional space, reducing the complexity of calculation and obtaining the optimal solution by calculating the partial differentiation of each feature using a Lagrange multiplier. As a well-liked kernel function, the radial basis function (RBF) kernel function was chosen as it has advantages in handling nonlinear relationships between spectral data and the target category [33]. A 10-fold cross-validation coupled with grid-search method was employed to search the optimal parameter values of regularization parameter gamma (*γ*) and kernel function parameter sig2 (*σ*2). PLS-DA is a supervised linear classifier based on PLS regression, which predicts the membership of the dataset by maximizing the covariance between the data matrix *X* (the maize spectral matrix) and the categorical *Y* matrix (sample labels). The predicted results obtained by PLS are continuous values, not strictly sample labels. Therefore, it is necessary to set a threshold to determine the prediction results of the model. The threshold was set as 0 in this study. In addition, a 10-fold cross validation was employed on the training dataset, followed by the determination of optimal latent variables according to the minimum classification accuracy. KNN is a statistical method for pattern recognition by tuning the hyperparameters, including distance metric and number of neighbors (K), which were optimized automatically to minimize 10-fold cross-validation loss [34]. ELM is a fast machine-learning algorithm proposed by Huang et al. [35], which shows high efficiency, rapidity, and good generalization performance in analyzing large-scale spectral data. The number of hidden neurons in ELM was increased from 10 to 150 with a step of 10, followed by the determination of the optimal hidden neurons according to the classification results.

### 2.6. Software

In this study, the spectral data analysis was performed in MATLAB 2018b (The Math Works, Natick, MA, USA) with the assistance of libpls toolbox [36]. Origin 2018 (Origin Lab Corporation, Northampton, MA, USA) was applied to construct figures.

## 3. Results and Discussion

### 3.1. Features of Spectra

**Figure 2:**
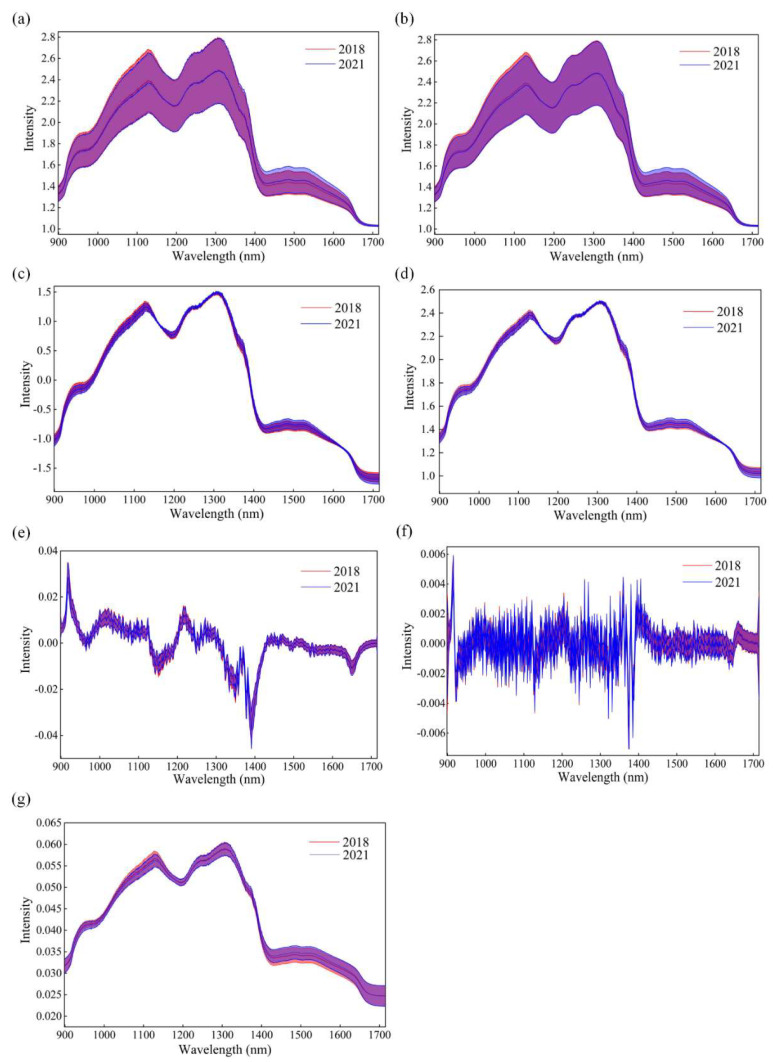
The average spectra (solid line) with their standard deviation (shadow region). (**a**) The original spectra curves; (**b**–**g**) stand for the spectra preprocessed by SGS, SNV, MSC, SG-D1, SG-D2, and Norm.

The tested sample is irradiated with near-infrared radiation, with transmitted or reflected radiation measured by a spectrometer. The spectral characteristics change through scattering and absorption processes and can reflect the structure and content information of X-H groups (C–H, O–H and N–H) in the sample [37]. Figure 2a shows the raw average spectra (solid line) and deviation distribution (shadow region) of the maize seeds. The blue part represents the seeds harvested in 2021 and the red part represents the seeds harvested in 2018. It can be clearly seen that the spectra of new and aged maize seeds have similar intensity and trend. The prominent absorption peak and valley were located at around 1110 nm, 1200 nm, and 1300 nm. The spectral intensity peak and valley at about 1110 nm and 1200 nm might be relative to the second overtone of C-H [38], while the peak at around 1300 nm is possibly caused by the combination of the fundamental amide vibrations and the first overtone of Amide B [39]. It is difficult to directly distinguish seeds in different harvest years by only depending on spectral intensity. Therefore, it is necessary to establish an appropriate classification model using machine learning and chemometric methods.

### 3.2. Classification Results Using Full Spectra

Figure 2b–g represent the spectra pretreated by SGS, SNV, MSC, SG-D1, SG-D2, and Norm, respectively. It can be seen that SGS effectively reduced the noise of the spectral data, while MSC and SNV eliminated the influence of scattering. However, the SG-D1 and SG-D2 preprocessing methods did not improve the spectral quality. It is worth noting that the spectral curves of the seeds harvested in different years after Norm pretreatment showed more differences. However, there were still obvious overlaps between the two types of spectra.

In order to determine the optimal pretreatment method, the spectral data preprocessed by SGS, SNV, MSC, SG-D1, SG-D2, and Norm were used as inputs to establish PLS-DA, LS-SVM, KNN, and ELM models. The key parameters (γ, σ^2^ for LS-SVM, LVs for PLS-DA, K for KNN, and hidden neurons for ELM) and classification results in the training set and the prediction set are summarized in Table 2. 

In comparison with the inspection results obtained by the raw spectra, the classification results yielded by the preprocessed spectra were improved to different degrees except for the model built with derivative spectra. Compared with other preprocessing methods, the spectra pretreated by SGS obtained the best results regardless of the classification methods. It can be seen that the result of the four classification models represented significant differences. Basically, PLS-DA obtained the best performance in inspecting seeds from different harvest years by comparing the four classification methods. When the number of LVs was equal to 7, 8, 8, 7, 5, and 10, the best discrimination results were obtained with spectral data preprocessed by SGS, SNV, MSC, SG-D1, SG-D2, and Norm preprocessing, respectively. The PLS-DA model based on spectra pretreated by Norm yielded relatively higher accuracy in discriminating between kernels from different harvest years, with classification accuracies of 96.56% and 91.25%, respectively for the training and prediction sets. Therefore PLS-DA will be used for further analysis. The above classification results indicated that near-infrared spectroscopy technology can achieve the on-line recognition of new and aged maize seeds. Seed quality properties, such as freshness, damage, and internal components, can be evaluated simultaneously through an on-line NIR device and multiple NIR calibration models, requiring high computing performance and shorter measurement time, especially when the seeds are inspected at a fast speed. In addition, the full spectra contain redundancy and multicollinearity variables among contiguous wavebands, thus causing time-consuming calibration processes and hindering the computing speed. Thus, it is necessary to select effective wavelengths to simplify calibration models.

### 3.3. Classification Results Based on Effective Wavelengths

At first, four groups of effective wavelengths selected by MC-UVE, CARS, BOSS, and SPA were separately used as the inputs to establish PLS-DA models for identifying maize seeds harvested in different years. The discrimination results of the PLS-DA models built with effective wavelengths selected by different wavelength selection methods are shown in Table 3.

When the numbers of LVs were equal to 8, 7, 7, and 6, the best classification results were acquired by MC-UVE-PLS-DA, CARS-PLS-DA, BOSS-PLS-DA, and SPA-PLS-DA, respectively, with classification accuracies of 86.25, 81.25, 78.75, and 70.00%. The classification accuracy of CARS-PLS-DA and BOSS-PLS-DA models on the prediction set were 81.25% and 78.75%, respectively. It can be clearly seen that SPA and MC-UVE-SPA could effectively compress data, with the selected number of wavelengths of 6 and 4, respectively, but the classification accuracy of the prediction set was smaller than 70%. The MC-UVE-PLS-DA model’s performance was better than the CARS-PLS-DA model, with success rates of 90% and 82.5% for aged and new seeds, respectively. MC-UVE-PLS-DA obtained more promising classification results for seeds from different harvest years. However, the number of selected wavelengths was a little higher, and it can be reduced further by using other variable selection methods. MC-UVE-SPA-PLS- DA and MC-UVE-BOSS-PLS-DA models were then developed, respectively. The MC-UVE-BOSS-PLS-DA model obtained the optimal performance with an accuracy of 88.75% for the prediction set. Compared with the full-spectrum PLS-DA model, the MC-UVE-BOSS-PLS-DA model reduced the classification accuracy of prediction sets by 2.5%. However, the MC-UVE-BOSS-PLS-DA model only used 93 wavelengths (18% of full spectral data). It is worth noting that the MC-UVE-BOSS-PLS-DA model has a prediction accuracy of 92.5% for new seeds, which is the same accuracy as the full-spectrum PLS-DA model. The results demonstrated that the NIR spectra collected from the on-line acquisition device could establish a classification model for detecting maize seeds from different harvest years. The optimal models developed by 93 effective wavelengths were more suitable for on-line application.

### 3.4. Wavelength Selection Analysis of the Optimal Models

**Figure 3:**
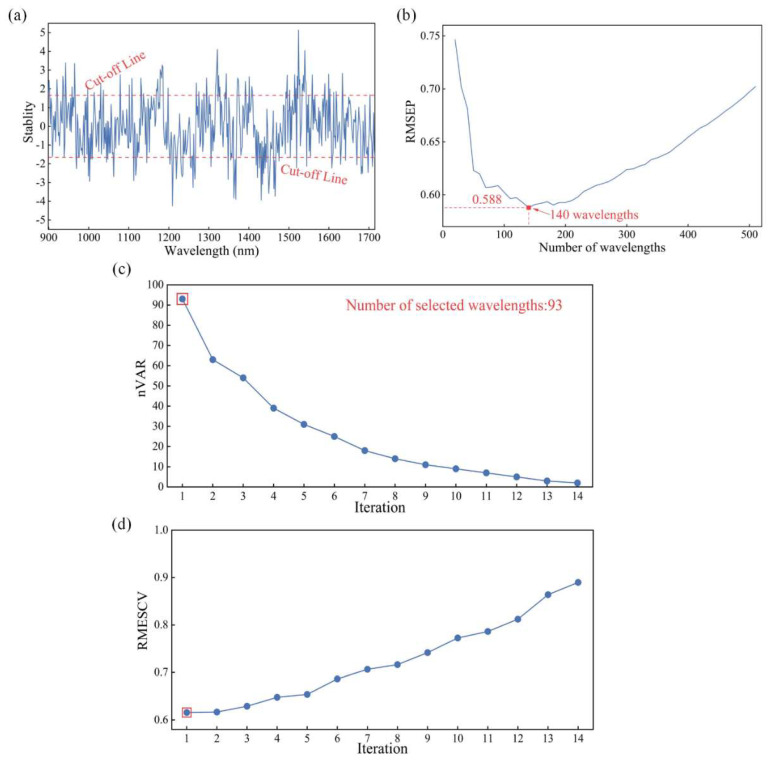
Feature wavelength selection for seeds harvested in different years by MC-UVE-BOSS from NIR spectra after pretreatment by Norm. (**a**) The stability of full wavelengths, (**b**) RMSEP variation with the number of selected wavelengths, (**c**) the variation in the number of variables (nVAR) and (**d**) RMSECV in sub-models in each iteration of BOSS.

As discussed in Section 3.4, MC-UVE-BOSS, a combination of MC-UVE and BOSS, was an effective wavelength selection method in the identification of maize seeds from different harvest years. Figure 3 shows the wavelength selection process of the MC-UVE-BOSS method. In the process of MC-UVE, 320 samples in the training set were applied as the input for the MC-UVE algorithm for selecting effective wavelengths. The stability of each wavelength was calculated and is shown in Figure 3a. The absolute values of stability were sorted in a descending manner. A number (N) of the variables were then selected from the sorted stabilities as informative wavelengths, corresponding to the stability of the Nth variable as the cutoff value. As the N increased from 20 to 500 with a step of 20, a set of PLS models were developed and applied to the prediction set, followed by the calculation of RMSEP values. Figure 3b represents the RMSEP variation of the PLS calibration model with the increase in the number of variables. It can be seen that the lowest RMSEP value was acquired when the number of the selected wavelengths was 140, corresponding to the wavelength’s stability outside the cut-off line (Figure 3a, red dotted line). 

**Figure 4:**
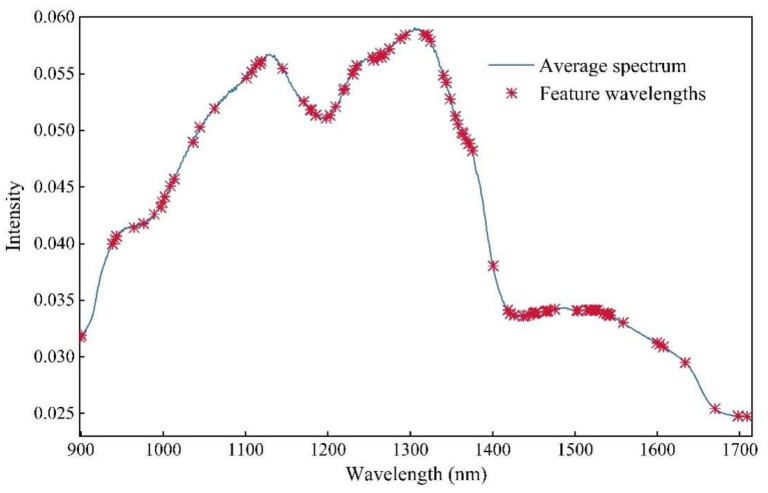
The distribution of feature wavelengths selected by MC-UVE-BOSS.

After wavelength selection by the MC-UVE method, the BOSS method was used to further extract effective information. In the process of BOSS, 140 wavelengths selected by MC-UVE were used to generate a large number of subsets in variable space, which were used to build PLS sub-models. The sub-models with a smaller RMSECV were extracted (10%), obtaining the regression coefficients and then new weights for the variables. Based on the new weights, a weighted bootstrap sampling (WBS) method was used to generate a new subset. The number of selected variables decreased gradually and reached 1 after 14 iterations (Figure 3c). Meanwhile, the RMSECV in the sub-models increased during the iterations. It can be seen that the minimum RMSECV value was obtained at the first iteration, with the number of selected wavelengths of 93. The wavelengths selected by MC-UVE-BOSS were mainly located at 1000–1020, 1260–1300, 1420–1480, and 1500–1520 nm (Figure 4). The wavelengths near 1200 nm were associated with the attributes of the secondary stretching vibration of C–H bonds in starch, proteins, or lipids [40]. The selected wavelengths around 1400–1500 nm were the absorption wavelengths of the first overtone of O-H and N-H stretching, which were related to the water and protein contents of maize seeds [41]. The wavelengths near 1700 nm were relevant to the absorption wavelength of the first overtone of C-H, which was caused by the strong absorption of fatty acids contained in the maize kernels [42]. This indicates that the selected wavelengths contain rich information related to the nutritional composition of maize seeds.

## 4. Conclusions

An on-line NIR device with a spectral range of 899–1715 nm was successfully utilized for the non-destructive and rapid classification of maize seeds harvested in different years. Four classic classification models, including PLS-DA, LS-SVM, KNN, and ELM, were established based on full spectra (512 wavelengths). The results indicated that the PLS-DA was more suitable for distinguishing maize seeds harvested in different years. The norm method has been determined as the optimal preprocessing method based on the PLS-DA model. In order to further improve the performance of the model, different wavelength selection methods were adopted. The classification accuracy of MC-UVE-BOSS-PLS-DA for the maize seed harvest year was 88.75%, which was slightly lower than the 91.25% achieved by the full-spectrum PLS-DA model. However, the number of wavelengths used by the MC-UVE-BOSS-PLSDA model was only about 1/6 of the full-spectrum PLS-DA model. Moreover, the classification accuracy of new seeds by the MC-UVE-BOSS-PLSDA model was equal to the full-spectrum PLS-DA model. In the next step, improvements in the classification accuracy will be studied by optimizing equipment and spectral data methods, thereby promoting the on-line application of this technology.

## Figures and Tables

**Table 1:** Class assignment and partition of sample sets.

Sample Class	No. of Samples	Training Set	Prediction Set	Assigned Class
Seeds harvested in 2018	200	160	40	−1
Seeds harvested in 2021	200	160	40	1

**Table 2:** The discrimination results for seeds harvested years by different classification models and preprocessing methods using full wavelengths.

Models	Preprocessing Methods	(*γ*, *σ*2)	LVs	K	Hidden Neurons	Classification Accuracy of Training Set (%)			Classification Accuracy of Prediction Set (%)		
						2018	2021	Total	2018	2021	Total
LS-SVM	Raw	59,845.85; 14,662.89				100.00	99.38	99.69	82.50	87.50	85.00
	SNV	18,298.45; 11,9622.43				98.75	100.00	99.38	82.50	85.00	83.75
	MSC	1280.47; 43,116.50				95.00	95.63	95.31	85.00	87.50	86.25
	Norm	4062.37; 7016.89				100.00	100.00	100.00	92.50	87.50	90.00
	SGS	9440.09; 317.76				100.00	100.00	100.00	97.50	80.00	88.75
	S-G-1st	91.37; 4736.06				93.13%	92.50	92.81	77.50	80.00	78.75
	S-G-2nd	10,870.69; 52,589.62				97.50	97.50	97.50	77.50	67.50	72.50
PLSDA	Raw		9			98.13	97.50	97.81	87.50	85.00	86.25
	SNV		7			98.13	93.75	95.94	90.00	90.00	90.00
	MSC		8			98.75	97.50	98.13	90.00	85.00	87.50
	Norm		8			98.13	95.00	96.56	90.00	92.50	91.25
	SGS		7			86.88	87.50	87.19	90.00	85.00	87.50
	S-G-1st		5			96.88	98.13	97.50	70.00	75.00	72.50
	S-G-2nd		10			100.00	100.00	100.00	70.00	77.50	73.75
KNN	Raw			4		87.50	69.38	78.44	77.50	62.50	70.00
	SNV			10		73.75	69.38	71.56	65.00	67.50	66.25
	MSC			5		74.38	73.75	74.06	72.50	67.50	70.00
	Norm			8		93.75	92.50	93.13	90.00	85.00	87.50
	SGS			6		93.75	95.63	94.69	90.00	82.50	86.25
	S-G-1st			1		100.00	100.00	100.00	65.00	70.00	67.50
	S-G-2nd			5		73.13	75.00	74.06	67.50	57.50	62.50
ELM	Raw				140	85.13	83.70	84.41	73.40	68.75	71.08
	SNV				60	81.25	81.25	81.25	77.50	85.00	81.25
	MSC				100	86.25	82.50	84.38	82.50	80.00	81.25
	Norm				90	100.00	100.00	100.00	87.50	92.50	90.00
	SGS				110	98.13	99.38	98.75	92.50	82.50	87.50
	S-G-1st				50	80.00	77.50	78.75	77.50	82.50	80.00
	S-G-2nd				60	80.63	78.75	79.69	72.50	70.00	71.25

**Table 3:** The classification results for PLS-DA models using different wavelength selection methods.

Model	No. of Wavelengths	LVs	Classification Accuracy of Training Set (%)			Classification Accuracy of Prediction Set (%)		
			2018	2021	Total	2018	2021	Total
MC-UVE-PLS-DA	140	8	96.88	96.25	96.56	90.00	82.50	86.25
CARS-PLS-DA	48	7	93.13	92.50	92.81	82.50	80.00	81.25
BOSS-PLS-DA	107	7	96.88	95.00	95.94	80.00	77.50	78.75
SPA-PLS-DA	6	6	61.25	71.88	66.56	67.50	72.50	70.00
MC-UVE-SPA-PLS-DA	4	4	59.38	73.13	66.25	62.50	72.50	67.50
MC-UVE-BOSS-PLS-DA	93	8	95.63	94.38	95.00	85.00	92.50	88.75

## Data Availability

The original contributions presented in the study are included in the article, further inquiries can be directed to the corresponding author.
